# Sexual Function After Corrective Spinal Osteotomy in Female Patients with Ankylosing Spondylitis: A Retrospective Single-Center Case Series

**DOI:** 10.3390/jcm15207997

**Published:** 2026-10-15

**Authors:** Muhammed Fatih Serttas, Alperen Uysal, Alper Kurtoglu, Nazim Canberk Kilic, Zeki Ceran Eraslan, Fevzi Saglam, Mustafa Erkan Inanmaz

**Affiliations:** 1 Department of Orthopaedics and Traumatology, Faculty of Medicine, Marmara University, Istanbul 34854, Türkiye; opdrnckilic@gmail.com (N.C.K.); zekiceran32@gmail.com (Z.C.E.); 2 Department of Orthopaedics and Traumatology, Faculty of Medicine, Sakarya University, Sakarya 54290, Türkiye; uysalalperen@yahoo.com (A.U.); dralperkurtoglu@gmail.com (A.K.); fevzisaglam@sakarya.edu.tr (F.S.); erkaninanmaz@yahoo.com (M.E.I.)

**Keywords:** ankylosing spondylitis, pedicle subtraction osteotomy, sexual function, sagittal balance, Female Sexual Function Index

## Abstract

**Background/Objectives:** Ankylosing spondylitis (AS) may cause progressive spinal rigidity and kyphotic deformity, with consequences for physical function, quality of life, and sexual health. Data regarding sexual function following corrective spinal osteotomy in women with AS are limited. This study aimed to describe radiological and functional changes and to explore within-patient changes in sexual function following corrective spinal osteotomy. **Methods:** This retrospective single-center case series analyzed prospectively maintained data from female patients with AS who underwent single-level pedicle subtraction osteotomy for thoracolumbar kyphosis between 2015 and 2024. The primary outcome was the change in total Female Sexual Function Index (FSFI) score from baseline to the standardized two-year assessment. Secondary outcomes (analyzed as exploratory, without adjustment for multiplicity) included FSFI domains, Visual Analog Scale, Bath Ankylosing Spondylitis Functional Index, and radiological parameters. Sexual-activity frequency, time to resumption of sexual activity, and preferred coital position were also recorded. Paired outcomes were analyzed using the exact Wilcoxon signed-rank test, and exploratory radiological–FSFI associations using Spearman’s correlation with false-discovery-rate adjustment. **Results:** Eleven patients with complete two-year data were included. Significant postoperative changes were observed in several sagittal alignment parameters, as well as in Visual Analog Scale (VAS) and Bath Ankylosing Spondylitis Functional Index (BASFI) scores. Total FSFI and the desire, arousal, orgasm, and satisfaction domains increased, whereas lubrication and pain did not change significantly. A descriptive shift toward higher-frequency sexual activity and different preferred coital positions was also observed. Radiological–FSFI correlations were interpreted cautiously because of the small sample and multiple comparisons. **Conclusions:** In this small exploratory cohort, postoperative assessment was associated with improved radiological alignment, pain, physical function, and several domains of self-reported sexual function. These findings represent within-patient changes and exploratory associations and do not establish a causal effect of spinal osteotomy on sexual function or a specific safe interval for resumption of sexual activity. Larger prospective studies are needed to confirm these observations.

## 1. Introduction

Ankylosing spondylitis (AS) is a systemic, chronic, and progressive inflammatory disease that primarily involves the sacroiliac joints and the spine [1,2]. In addition to axial involvement, affection of major peripheral joints such as the shoulder and hip further increases functional impairment [2,3]. In advanced stages, rigidity and kyphotic deformity disrupt sagittal balance, thereby limiting patients’ functional capacity and activities of daily living. Beyond its impact on daily functioning, AS may also adversely affect sexual function. Previous studies have demonstrated that sexual dysfunction is more prevalent among patients with ankylosing spondylitis and axial spondyloarthritis and may be associated with pain, functional limitations, disease-related factors, and psychosocial variables [4,5]. Clinically, sexual dysfunction may present as decreased libido, difficulty with arousal, orgasmic disorders, and pain during intercourse.

Spinal osteotomy is an effective surgical technique for restoring sagittal balance in patients with AS who have severe kyphotic deformity [6]. Although several studies have evaluated the impact of AS on sexual function [7], investigations examining the effect of radiological correction achieved after spinal osteotomy on sexual function are extremely limited [8]. In particular, the postoperative return to sexual activity and changes in sexual function among female patients with AS have not been adequately explored.

The aim of the present study was to describe changes in radiological and functional outcomes and to explore within-patient changes in sexual function, sexual-activity frequency, and preferred sexual positions over the two-year postoperative period in a cohort of female patients with AS who underwent corrective spinal osteotomy for kyphotic deformity.

## 2. Materials and Methods

### 2.1. Study Design and Patient Selection

This study was designed as a retrospective analysis of a prospectively maintained single-center database of female patients with ankylosing spondylitis (AS) evaluated for corrective spinal osteotomy between 2015 and 2024. Following approval by the institutional ethics committee, patient records and prospectively collected clinical, radiological, and sexual-function data were retrospectively reviewed. All consecutive female patients with AS evaluated for corrective spinal osteotomy during the study period were screened for eligibility.

The inclusion criteria were as follows:

- Diagnosis of AS according to the modified New York criteria [9];
- Presence of kyphosis in the thoracolumbar and/or lumbar spine;
- Female sex;
- Age between 18 and 50 years;
- Premenopausal status;
- Having an active sexual life with a regular partner prior to surgery.

The exclusion criteria were as follows:

- Cervicothoracic kyphosis;
- Presence of a preoperative neurological deficit.

A total of 17 female patients with AS who were evaluated for corrective spinal osteotomy during the study period were screened for eligibility. Three of these patients were excluded at the preoperative eligibility assessment: two had cervicothoracic kyphosis and therefore did not meet the inclusion criterion of thoracolumbar and/or lumbar kyphosis, and one had a preoperative neurological deficit, which was a predefined exclusion criterion. The remaining 14 patients fulfilled all eligibility criteria, underwent single-level PSO, and constituted the surgical and safety cohort. Of these 14 patients, one underwent revision surgery after the index procedure, and two patients developed transient postoperative neurological deficits that prolonged hospitalization and rehabilitation. These three patients were retained in the overall surgical cohort and included in safety reporting but did not have complete two-year sexual-function assessments and were therefore considered to have missing outcome data rather than being excluded from the surgical cohort. The remaining 11 patients had complete evaluable outcome data at the standardized two-year postoperative assessment and constituted the primary paired-outcome analysis population.

### 2.2. Surgical Procedure

All surgical planning was performed by an experienced spine surgeon, taking into account the severity of the deformity and sagittal balance parameters, and all procedures were carried out by the same surgical team. To correct the kyphotic deformity, single-level pedicle subtraction osteotomy (PSO) was performed via a posterior approach in all patients. PSO was performed at L3 in 7 patients and at L4 in 4 patients, depending on the severity of the deformity and preoperative sagittal alignment parameters. All patients received standardized postoperative counseling regarding resumption of sexual activity. They were advised that sexual activity could be resumed from the fourth postoperative week provided that their clinical recovery was satisfactory and no individual contraindication was present. The actual time to first postoperative sexual activity was subsequently recorded during routine follow-up.

### 2.3. Clinical and Radiological Assessment

Demographic and clinical data of the patients, including age, body mass index (BMI), and follow-up duration, were recorded and analyzed. Clinical evaluations were performed during routine postoperative outpatient follow-up visits. Standing whole-spine anteroposterior and lateral radiographs were obtained preoperatively and at the standardized two-year postoperative follow-up. Although patients continued to be followed clinically beyond the two-year assessment, the two-year postoperative time point was selected as the standardized assessment point for the present study in order to provide a uniform interval for comparison of preoperative and postoperative radiological, functional, and sexual-function outcomes. This time point was chosen to assess outcomes after the early postoperative recovery and rehabilitation period while maintaining a sufficiently standardized interval across patients. Radiographic measurements were performed using Sectra IDS7 software (Linköping, Sweden). Global kyphosis (GK) was measured as the angle between the superior endplate of T5 and the inferior endplate of S1. Lumbar lordosis (LL) was measured between the superior endplate of L1 and the superior endplate of S1. Thoracic kyphosis (TK) was measured between the superior endplate of T5 and the inferior endplate of T12. Pelvic incidence (PI) was defined as the angle between the perpendicular to the sacral endplate at its midpoint and the line connecting this point to the center of the femoral heads. Pelvic tilt (PT) was defined as the angle between the vertical reference line and the line connecting the center of the femoral heads to the midpoint of the sacral endplate. Sacral slope (SS) was defined as the angle between the superior endplate of S1 and the horizontal reference line. Sagittal vertical axis (SVA) was measured in millimeters as the horizontal distance between the C7 plumb line and the posterior superior corner of S1. The osteotomized vertebral angle (OVA) was defined as the angle formed by the superior and inferior endplates of the osteotomized vertebra. Preoperatively, the OVA was expected to approximate zero because the osteotomized vertebra was not yet subjected to angular correction. The small negative and positive preoperative values reflected measurement variability and/or the specific geometric definition of the angle. All radiographic measurements were performed by one observer with clinical experience. The observer was blinded to the clinical and sexual-function outcomes. Within the scope of clinical evaluation, back pain severity was assessed using the Visual Analog Scale (VAS), while functional status was evaluated using the Bath Ankylosing Spondylitis Functional Index (BASFI) [10,11]. Sexual function was assessed using the 19-item Female Sexual Function Index (FSFI), a validated patient-reported outcome measure comprising six domains: desire, arousal, lubrication, orgasm, satisfaction, and pain [12]. Higher scores indicate better sexual function in each domain, including the pain domain, for which a higher score indicates less pain during sexual activity. The Turkish-language validated version of the FSFI was administered to all participants. The questionnaire was self-administered by the participant in a private setting, without interviewer assistance, to ensure confidentiality of the sensitive information collected. Baseline sexual-function data were obtained prospectively before surgery. Postoperative FSFI data were obtained at the standardized two-year follow-up visit. In addition to the FSFI, participants were asked about the frequency of sexual activity, the time elapsed before resumption of sexual activity after surgery, and their preferred coital position. Preoperative sexual-activity frequency and preferred-position data were collected prospectively before surgery. Postoperative sexual-activity frequency and preferred-position data were collected at the same standardized two-year postoperative assessment. The time to resumption of sexual activity was recorded separately during routine postoperative follow-up visits and represents the interval reported by the patient between surgery and the first postoperative sexual activity. Sexual-activity frequency was categorized as 1–4, 5–8, 9–12, or >12 episodes per month. Preferred coital position was assessed using five illustrated options selected a priori to represent commonly reported positions: A—missionary (male superior, face-to-face), B—Amazon (female superior, face-to-face), C—spooning (male posterior, lateral decubitus), D—seated (face-to-face sitting position), and E—doggy style (male posterior, kneeling position) (Figure 1). Participants were asked to select only one position as their preferred coital position. The position-choice instrument was developed for the present study and was not a formally validated questionnaire. Because these measures were descriptive and the sample size was small, no causal or confirmatory inference was made from the observed changes in sexual-activity frequency or preferred position. Although the mean clinical follow-up was 58.9 months, all outcome measures used in the present analysis—including the FSFI, VAS, BASFI, and radiological parameters—were evaluated preoperatively and at a standardized 2-year postoperative follow-up. The time to resumption of sexual activity was recorded during routine interim postoperative follow-up visits.

### 2.4. Statistical Analysis

Statistical analyses were performed using SPSS Statistics 27 (IBM Corp., Armonk, NY, USA). Because of the small sample size, continuous variables were summarized using both mean ± standard deviation and median with interquartile or minimum–maximum ranges, as appropriate, and categorical variables were presented as counts and percentages. The primary outcome of the present analysis was the change in total Female Sexual Function Index (FSFI) score between baseline and the standardized two-year postoperative assessment. This designation was made retrospectively for the purpose of analytical transparency because no single primary outcome had been prospectively prespecified in the original study protocol. Changes in individual FSFI domains, VAS, BASFI, and radiological parameters were considered secondary outcomes; these secondary outcomes were analyzed in an exploratory manner, no adjustment for multiple comparisons was applied to them, and their *p* values are therefore reported as nominal values that should not be interpreted as confirmatory. Analyses of sexual-activity frequency, preferred coital position, and associations between radiological and FSFI changes were considered exploratory. All paired analyses were restricted to the 11 patients with complete preoperative and two-year postoperative data (complete-case analysis); no imputation was performed for missing outcome data. For paired continuous outcomes, the Wilcoxon signed-rank test was used. Because of the small sample size, exact two-sided *p* values were calculated. Effect sizes were calculated as r = Z/√N, where N is the number of paired observations (*N* = 11). For paired changes, 95% confidence intervals were estimated using a bias-corrected and accelerated (BCa) nonparametric bootstrap procedure with 10,000 resamples. Exact *p* values, effect sizes, and bootstrap 95% confidence intervals are reported for all paired outcomes, including the primary outcome (Tables 2, 4 and 5). Spearman’s rank correlation coefficient was used for exploratory associations between postoperative changes in radiological parameters and changes in FSFI domains. To account for multiple testing, the Benjamini–Hochberg false-discovery-rate (FDR) procedure was applied as a single family across all 56 correlations generated by eight radiological change variables and seven FSFI change variables. Nominal exact two-sided *p* values are shown in Table 6 for transparency; for correlations with nominal *p* < 0.05, the corresponding FDR-adjusted *p* values are reported in the Results, Figure 6, and Table 6 footnote. A correlation was regarded as statistically supported only if its FDR-adjusted *p* value was <0.05; all correlation analyses were otherwise interpreted as exploratory. Statistical significance for the primary paired comparison (total FSFI) was assessed at *p* < 0.05; the same threshold was applied only descriptively to the secondary paired comparisons, without correction for multiple testing. No formal a priori sample-size calculation was performed because of the rarity of the study population and the retrospective single-center case-series design. Accordingly, the present analysis should be regarded as exploratory and hypothesis-generating rather than confirmatory.

## 3. Results

Seventeen female patients with AS evaluated for corrective spinal osteotomy were screened for eligibility. Three patients were excluded at the preoperative eligibility assessment according to the predefined inclusion and exclusion criteria (cervicothoracic kyphosis, *n* = 2; preoperative neurological deficit, *n* = 1), and the remaining 14 eligible patients underwent single-level PSO. Of these, one patient required revision surgery and two developed transient postoperative neurological deficits; these three patients did not have complete two-year sexual-function assessments and were therefore retained in the surgical cohort and safety reporting but excluded from the primary paired-outcome analysis. The remaining 11 patients had complete evaluable two-year outcome data. The reasons for incomplete two-year outcome assessment are presented in Figure 2.

The mean age of the patients included in the study was 39.3 ± 6.3 years. The mean BMI was 25.6 ± 2.7 kg/m^2^. The mean instrumentation length was 8.8 ± 2.5 levels, and single-level PSO was performed at L3 in 7 patients and at L4 in 4 patients. The mean overall clinical follow-up duration was 58.9 ± 29.3 months (median, 62 months; range, 24–98 months). However, the primary clinical, radiological, and sexual-function outcomes analyzed in this study were assessed at a standardized two-year postoperative time point. The longer overall follow-up reflects continued clinical surveillance beyond the standardized research assessment. Among the 14 operated patients, one underwent revision surgery during follow-up, while two experienced transient postoperative neurological deficits. No other major implant-related complications were observed. The mean reported time to resumption of sexual activity was 7.9 ± 1.8 weeks among the patients with available data (Table 1). This value represents the observed time to resumption in this selected cohort and should not be interpreted as an independently established safe postoperative interval.

No statistically significant differences were observed between preoperative and postoperative PI and TK values. However, statistically significant improvements were detected in PT, SS, LL, GK, OVA, and SVA values (nominal *p* < 0.05; secondary outcomes, not adjusted for multiplicity) (Table 2).

Evaluation of monthly sexual activity frequency in the preoperative period revealed that 45.5% of patients reported engaging in sexual activity 1–4 times per month, 36.4% reported 5–8 times, 9.1% reported 9–12 times, and 9.1% reported more than 12 times per month. In the postoperative period, 18.2% of patients reported sexual activity 1–4 times per month, 45.5% reported 5–8 times, 27.3% reported 9–12 times, and 9.1% reported more than 12 times per month (Figure 3, Table 3). The distribution of sexual-activity frequency shifted descriptively toward higher-frequency categories at the postoperative assessment. Because of the paired nature of the observations and the small sample size, these findings are presented primarily using individual patient counts and transition patterns rather than percentages alone. The number of patients in each preoperative-to-postoperative category is shown in Figure 4. Regarding preferred sexual positions, the proportion of patients preferring the Amazon position decreased from 18.2% preoperatively to 9.1% postoperatively. Preference for the missionary position decreased from 36.4% to 27.3%, while preference for the seated position decreased from 27.3% to 9.1%. In contrast, preference for the doggy style position increased from 9.1% preoperatively to 27.3% postoperatively, and preference for the spooning position increased from 9.1% to 27.3% (Figure 5, Table 3).

Among the 11 patients included in the paired analysis, postoperative VAS and BASFI scores were lower than preoperative scores (nominal *p* < 0.05; secondary outcomes) (Table 4). For the primary outcome, total FSFI increased from 23.63 ± 5.45 preoperatively to 29.94 ± 2.48 at the two-year assessment (mean change, 6.31 ± 3.87; exact *p* < 0.001; r = 0.885; 95% CI for the mean change, 4.19 to 8.55) (Table 5). Among the secondary FSFI domains, desire, arousal, orgasm, and satisfaction scores were nominally higher postoperatively, whereas lubrication and pain-domain scores did not show statistically significant changes; these domain-level comparisons were exploratory and not adjusted for multiplicity (Table 5). Exploratory correlation analyses were performed between changes in radiological parameters and changes in FSFI domains. Change (Δ) was defined as postoperative minus preoperative; therefore, more negative ΔGK values represented greater reduction in global kyphosis. At the nominal level, ΔGK was correlated with ΔFSFI satisfaction (Spearman’s ρ = 0.819, 95% CI 0.508–0.976, exact *p* = 0.002), and ΔOVA was correlated with ΔFSFI pain (Spearman’s ρ = 0.612, exact *p* = 0.045) (Figure 6, Table 6). After the Benjamini–Hochberg procedure was applied across all 56 correlations, neither association remained statistically significant (FDR-adjusted *p* = 0.112 and 0.967, respectively); both were therefore treated as non-confirmatory. Leave-one-out analyses showed that the nominal association between ΔGK and ΔFSFI satisfaction was not driven by a single observation (ρ range, 0.761–0.856; all nominal *p* < 0.05), whereas the nominal association between ΔOVA and ΔFSFI pain became non-significant after removal of several individual observations, further indicating fragility.

## 4. Discussion

Patients with AS frequently face a higher risk for sexual dysfunction, likely driven by progressive spinal rigidity, postural deformity, and associated pain or functional limitations [13,14,15]. Previous studies have reported a higher prevalence of sexual dysfunction in both male and female patients with AS compared to the general population. In female patients in particular, decreased sexual desire, difficulty with arousal, impaired orgasm, and reduced sexual satisfaction are the most prominent concerns. However, the majority of existing studies have primarily focused on disease activity, pain, and psychological factors, while largely overlooking the impact of spinal deformity itself and the effects of its surgical correction on sexual function [16,17,18]. In the present study, PI and TK did not demonstrate significant changes between the preoperative and postoperative periods. This lack of change in TK is not unexpected, as all osteotomies were performed at lumbar levels (L3 or L4), and the primary surgical objective was restoration of global sagittal alignment rather than direct correction of thoracic curvature. In contrast, statistically significant improvements were observed in dynamic sagittal parameters, including PT, SS, LL, GK, OVA, and SVA, suggesting that the surgical intervention achieved a measurable realignment of global sagittal parameters in our patients. Objective assessment of lumbosacral alignment provides an additional framework for interpreting postoperative changes in patients undergoing spinal surgery. Recent work using three-dimensional posturography has demonstrated measurable postoperative changes in sagittal-plane parameters, including lordotic and sacral inclination measures, while also highlighting the potential influence of dynamic and diurnal adaptation on postoperative alignment assessment [19]. Although these findings were obtained in a different surgical population, they support the concept that postoperative spinal alignment is dynamic and reflects both structural correction and subsequent functional adaptation. In the present cohort, the observed changes in lumbar lordosis, sacral slope, pelvic tilt, global kyphosis, and sagittal vertical axis are consistent with the intended restoration of sagittal alignment after corrective osteotomy. These changes may modify trunk orientation and the mechanical demands placed on the lumbopelvic region during daily activities. However, the present study was not designed to directly measure spinal loading or dynamic biomechanics, and therefore any relationship between radiographic alignment and sexual function remains hypothetical and requires confirmation using objective biomechanical assessment. Structural restoration of sagittal balance in individuals with AS could potentially influence not only pain and functional capacity but also nuanced aspects of daily living, including sexual function [8]. Hägg et al. evaluated the impact of fusion surgery on sexual outcomes and reported that patients experienced improved sexual life postoperatively, with women reporting better sexual function than men after surgery [20]. Similarly, Verbruggen et al. demonstrated postoperative improvement in the sexual domain of the Oswestry Disability Index (ODI) in patients who underwent anterior fusion [21]. Consistent with these trends, our preliminary results showed postoperative improvements in sexual function parameters, reported frequency of sexual activity, and satisfaction scores within this sample. When the FSFI domains were analyzed individually, nominally significant increases were observed in desire, arousal, orgasm, and satisfaction scores, whereas no statistically significant changes were detected in lubrication and pain domains; because these domain-level analyses were exploratory and not adjusted for multiplicity, they should be interpreted with caution. These findings support the hypothesis that the observed improvements following surgical correction might be primarily linked to psychophysical and functional adjustments, while hormonal and local physiological factors, such as vaginal lubrication, do not appear to be directly influenced by spinal surgery. Likewise, the absence of a significant change in the pain domain implies that dyspareunia may be multifactorial in origin and not solely attributable to spinal deformity. The statistically significant increase in total FSFI and in selected FSFI domains should be distinguished from clinical meaningfulness. For context, a post hoc analysis of a randomized, sham-controlled trial in premenopausal women with self-reported vaginal laxity reported study-specific FSFI domain thresholds of 0.7 for desire, 1.0 for arousal, 1.0 for lubrication, 0.5 for orgasm, 0.9 for satisfaction, and 0.1 for pain [22]. These values were derived at six months for a different condition and intervention and have not been validated in women with AS undergoing corrective spinal osteotomy; an established FSFI total-score or domain-specific MCID is not available for this population. We therefore did not apply these thresholds to classify patients as clinically improved. The magnitude of the observed changes is presented descriptively, and population-specific responder criteria will require larger prospective studies incorporating patient-reported global assessments of improvement.

In the exploratory correlation analysis, ΔGK showed a nominal positive association with change in FSFI satisfaction. Because ΔGK was calculated as postoperative minus preoperative, more negative values represented greater kyphosis correction; consequently, the direction of this association does not support the interpretation that greater correction produced greater improvement in satisfaction. The association also did not remain statistically significant after FDR adjustment. The nominal association between ΔOVA and ΔFSFI pain was likewise non-significant after FDR adjustment and was unstable in the leave-one-out analysis. Although altered sagittal alignment could theoretically change trunk mechanics, this study did not measure dynamic spinal loading, and the small, uncontrolled sample precludes a reliable mechanistic or causal interpretation of either correlation.

Data regarding the timing of resumption of sexual activity after spinal surgery remain limited. Previous studies have reported variable recommendations after different procedures, but these findings cannot be directly extrapolated to corrective osteotomy for AS. Akbaş et al. reported no significant change in sexual desire after lumbar disc herniation surgery but observed a statistically significant increase in the frequency of sexual intercourse postoperatively [23]. Yao et al. reported an increase in the proportion of male patients with AS-related kyphosis engaging in sexual activity five or more times per month after surgery [8]. In the present cohort, the mean reported time to resumption was approximately eight weeks. Because all patients had received counseling permitting resumption from the fourth postoperative week, this interval may reflect counseling, individual recovery, symptoms, and personal preference rather than an independently established safety threshold. Thomas et al. found no consensus among spine surgeons on specific postoperative intervals [24]. Other reports concern adolescent scoliosis, lumbar disc surgery, or total hip arthroplasty rather than corrective osteotomy in women with AS [25,26,27]. In particular, the 5–12-week interval reported by Fu et al. followed total hip arthroplasty in male patients with AS and should not be interpreted as a recommendation after spinal osteotomy [27]. No major implant-related complication temporally attributed to sexual activity was identified in our series; however, adverse events were not evaluated using a protocol designed to assess sexual activity, and this observation cannot establish the safety of a particular postoperative interval.

The documented shifts in preferred sexual positions are exploratory and may reflect multiple factors, including pain, perceived comfort, functional limitations, partner preference, and adaptation to the postoperative posture. Keefe et al. reported difficulty with particular positions and postoperative changes in position among patients after cervical fusion [28]. Yao et al. found no statistically significant change in preferred positions among male patients with AS, although the most commonly reported position changed descriptively over time [8]. In our cohort, Amazon, missionary, and seated positions were more frequently reported preoperatively, whereas spooning and doggy style were more frequently reported postoperatively. These shifts do not demonstrate that any position produces lower spinal loading or is biomechanically safer. Sidorkewicz et al. showed that lumbar motion varies by sexual position in male participants and that position-specific recommendations depend on the direction of motion intolerance [29]; those findings cannot be directly generalized to women with a rigid AS spine after long-segment fixation and osteotomy. Because we did not measure spinal motion, implant loading, pain during specific positions, or position-related adverse events, no position-specific biomechanical or safety recommendation can be made from the present data.

Horst et al., in their study of patients who underwent decompression and/or posterior fusion for spinal stenosis and degenerative spondylolisthesis, concluded that surgical treatment was associated with a lower incidence of pain during sexual activity at all follow-up time points [30]. In contrast, Yao et al. reported no statistically significant difference between preoperative and postoperative VAS scores in male patients with AS undergoing spinal surgery [8]. They attributed this finding to the natural course of the disease, suggesting that in the late stages of AS characterized by established kyphotic deformity, low back pain is often less severe compared to the earlier, more active inflammatory phase. In studies focusing on female patients with AS, pain has generally been evaluated as a marker of disease activity rather than as an independent clinical parameter. In our study, the significant reductions observed in VAS and BASFI scores point toward a notable decrease in pain and functional limitation following surgery within this cohort. Postoperative reductions in pain and functional limitation may be relevant contextual factors when interpreting the observed changes in sexual function. In this context, the relationship between postoperative spinal alignment and sexual function is unlikely to be explained by mechanical factors alone and may also involve functional and psychosocial factors. Beyond biomechanical realignment, improvements in overall posture, functional independence, and general well-being may contribute to psychosocial recovery, which could subsequently support postoperative sexual health.

This study has several important limitations. First, the uncontrolled before-and-after design and absence of a control group do not permit causal inference regarding the relationship between corrective spinal osteotomy and postoperative changes in sexual function. Second, the very small sample size limits statistical power, precision, and generalizability, and no formal a priori sample-size calculation was performed. Third, exclusion or incomplete outcome assessment after postoperative complications may have introduced selection and attrition bias, despite retention of these patients in the overall surgical and safety cohorts. Fourth, the large number of radiological and FSFI-related comparisons increased the risk of type I error; therefore, radiological–FSFI correlations were treated as exploratory and subjected to false-discovery-rate adjustment, whereas the secondary paired comparisons of FSFI domains, VAS, BASFI, and radiological parameters were not adjusted for multiplicity and should likewise be regarded as exploratory. Fifth, potentially important confounding variables, including disease activity, medication changes, hip involvement, psychological status, partner-related factors, rehabilitation, and menopausal transition, were not systematically assessed. Finally, sexual function was assessed at a single standardized postoperative time point, and the position questionnaire was not formally validated. These limitations support interpreting the present findings as exploratory and hypothesis-generating rather than confirmatory.

## 5. Conclusions

In this small, uncontrolled series of women undergoing corrective spinal osteotomy, two-year follow-up demonstrated improvements in sagittal alignment and functional outcomes, accompanied by higher total FSFI scores and improvements in selected FSFI domains. Owing to the limited sample size, lack of a control group, and potential residual confounding, these findings should be considered preliminary and hypothesis-generating. They do not establish a causal relationship or support specific recommendations regarding the timing or biomechanical safety of postoperative sexual activity. Nevertheless, the findings highlight sexual health as a potentially relevant component of perioperative assessment and support further investigation in larger, prospective controlled studies.

## Figures and Tables

**Figure 1:**
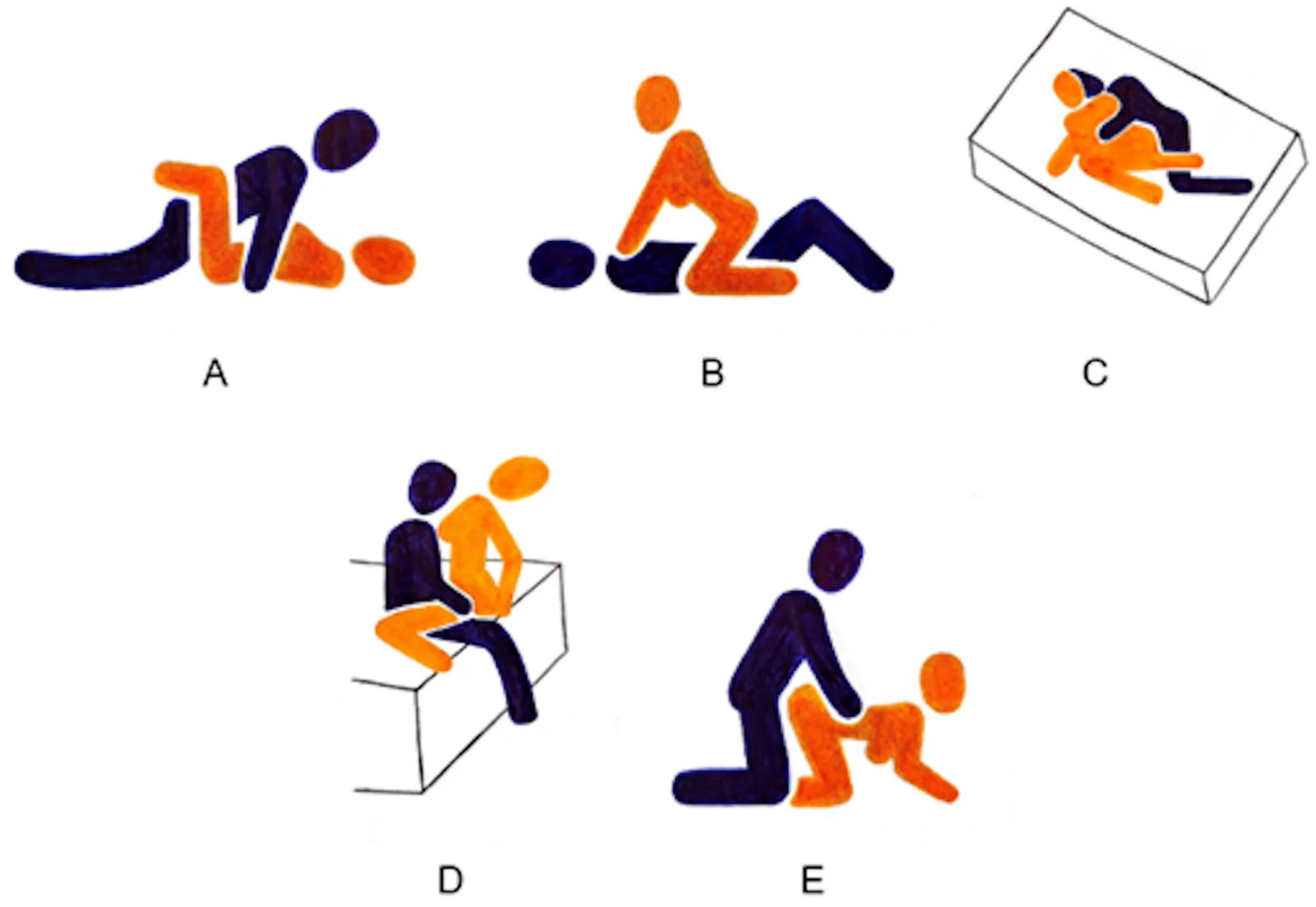
Common sexual positions used in this study. In all panels, the man is represented in blue and the woman is represented in orange. (**A**) missionary (male superior, face-to-face); (**B**) Amazon (female superior, face-to-face); (**C**) spooning (male posterior, lateral decubitus); (**D**) seated (face-to-face sitting position); (**E**) doggy style (male posterior, kneeling position).

**Figure 2:**
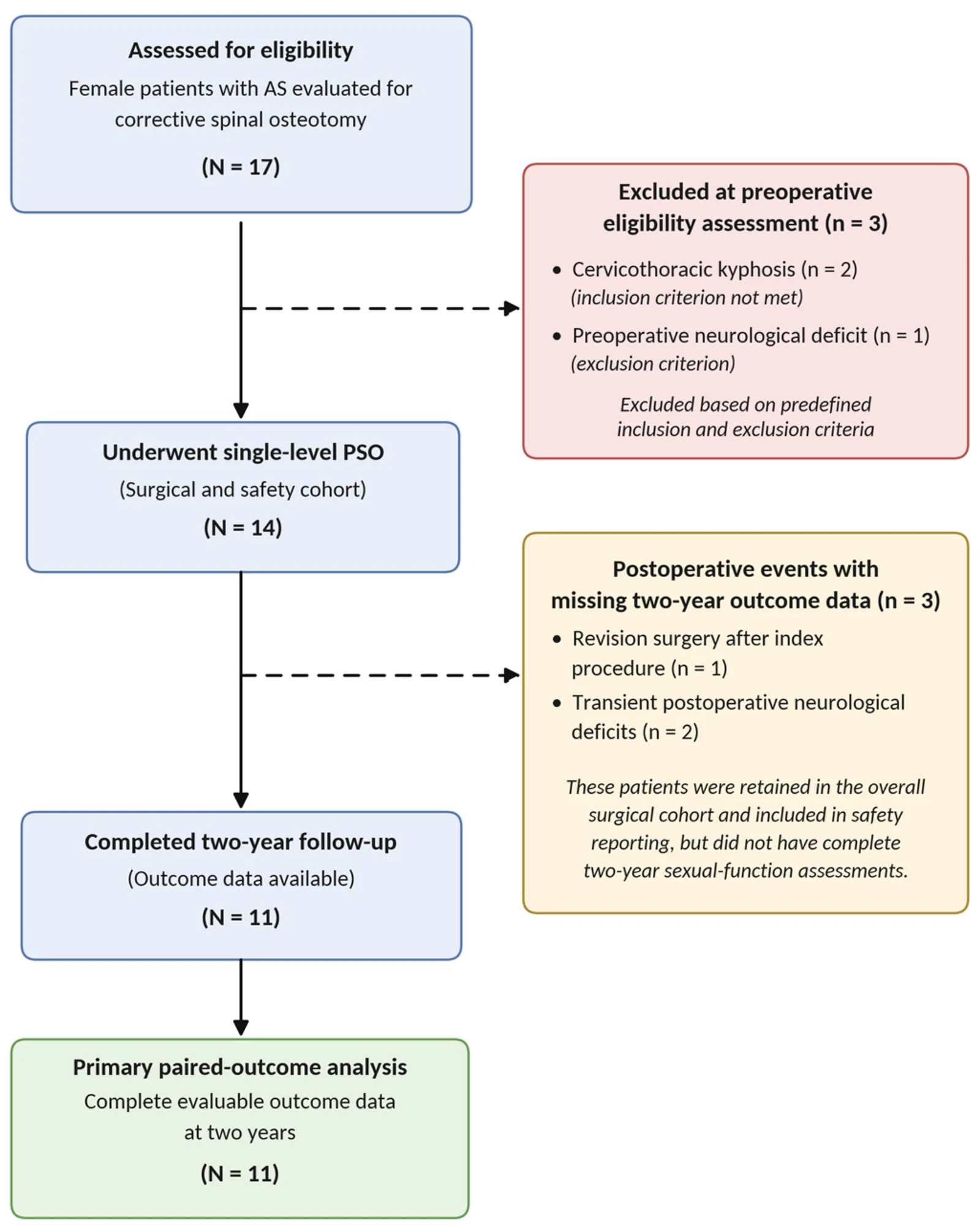
Participant flow diagram showing the screening, eligibility assessment, surgical treatment, postoperative events, availability of two-year follow-up data, and populations included in the primary outcome and safety analyses. Three patients were excluded at the preoperative eligibility assessment according to the predefined inclusion and exclusion criteria described in Section 2.1.

**Figure 3:**
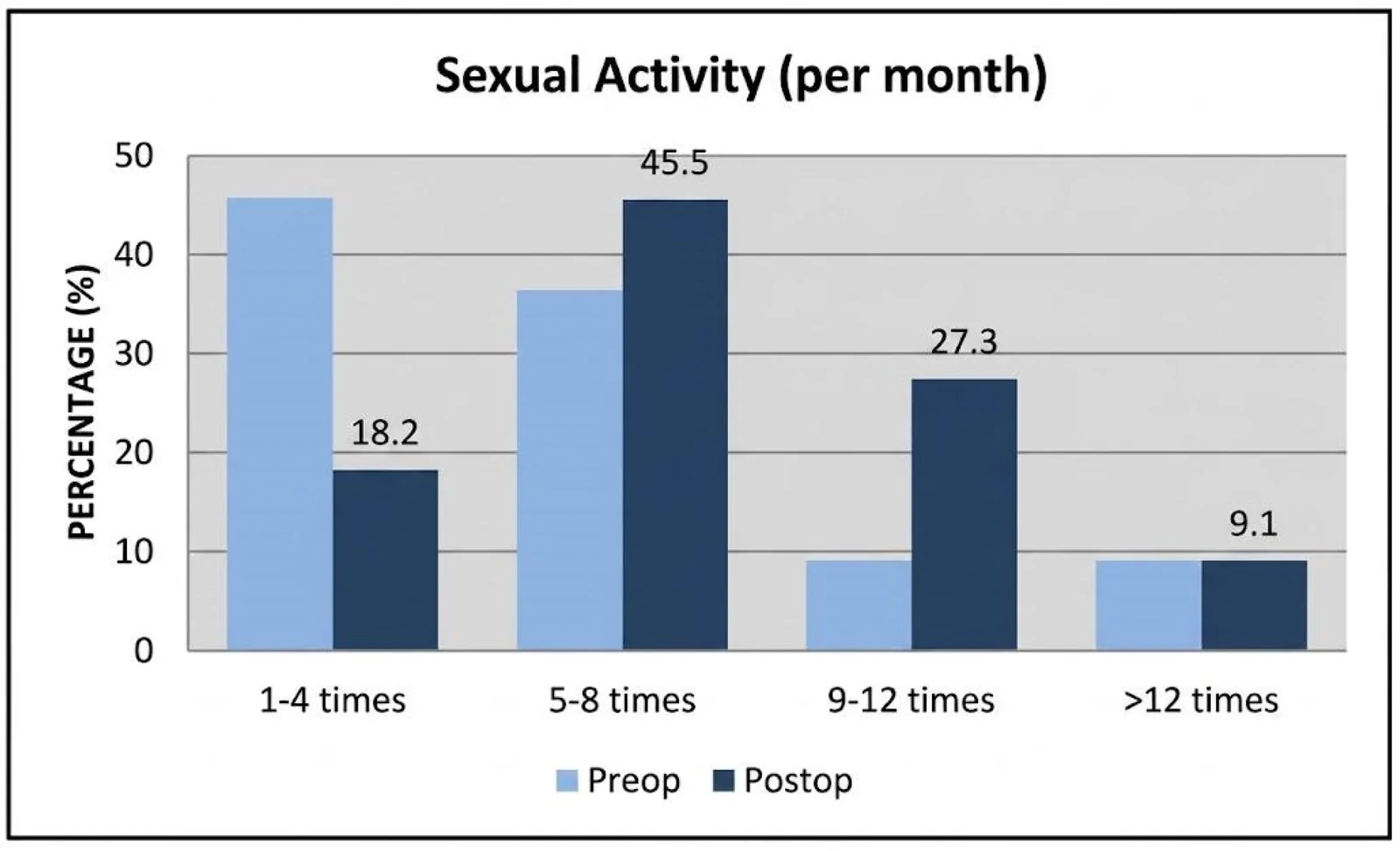
Frequency of sexual activity before and two years after surgery. Values are presented as the number of patients and percentages.

**Figure 4:**
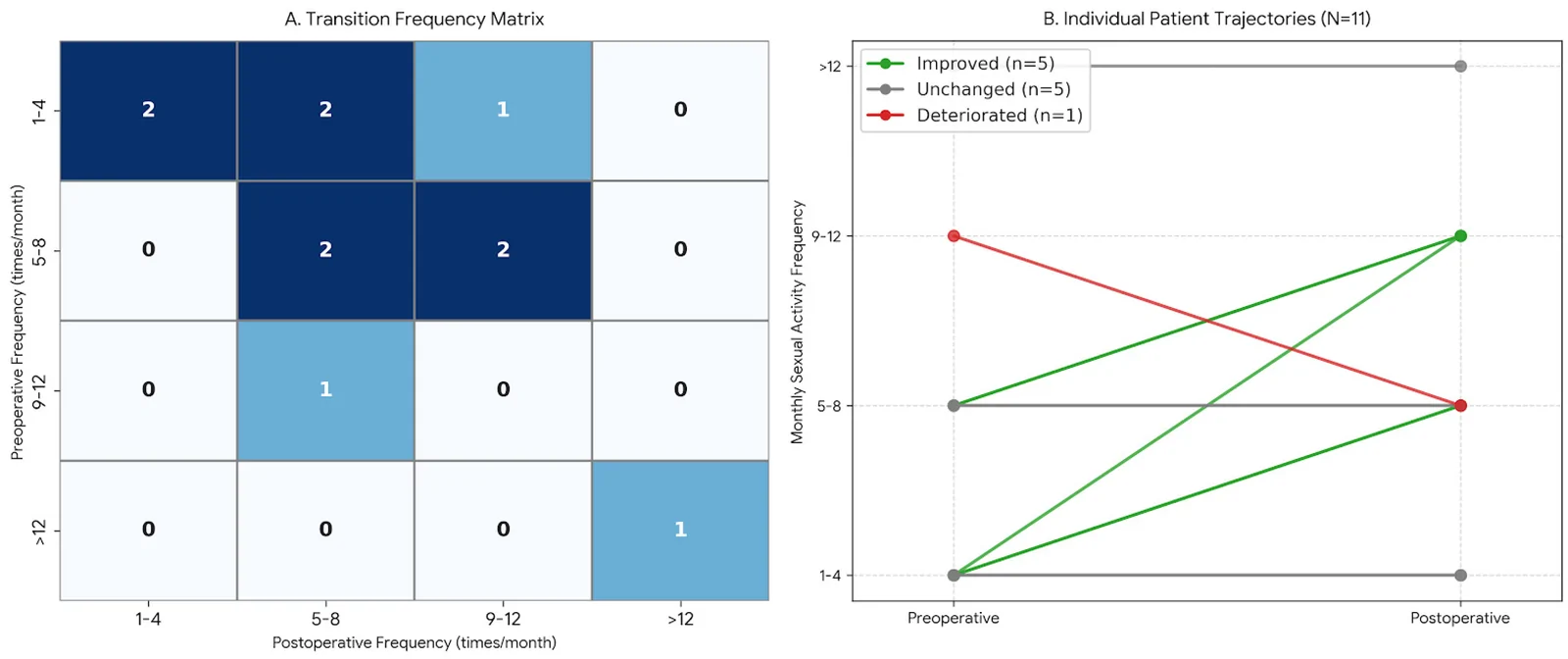
Preoperative to postoperative sexual activity frequency transition analysis. (**A**) Cross-tabulation heatmap displaying baseline versus postoperative monthly frequency categories. (**B**) Individual patient trajectories color-coded by postoperative outcome status: Improved (green, *n* = 5), Unchanged (grey, *n* = 5), and Deteriorated (red, *n* = 1).

**Figure 5:**
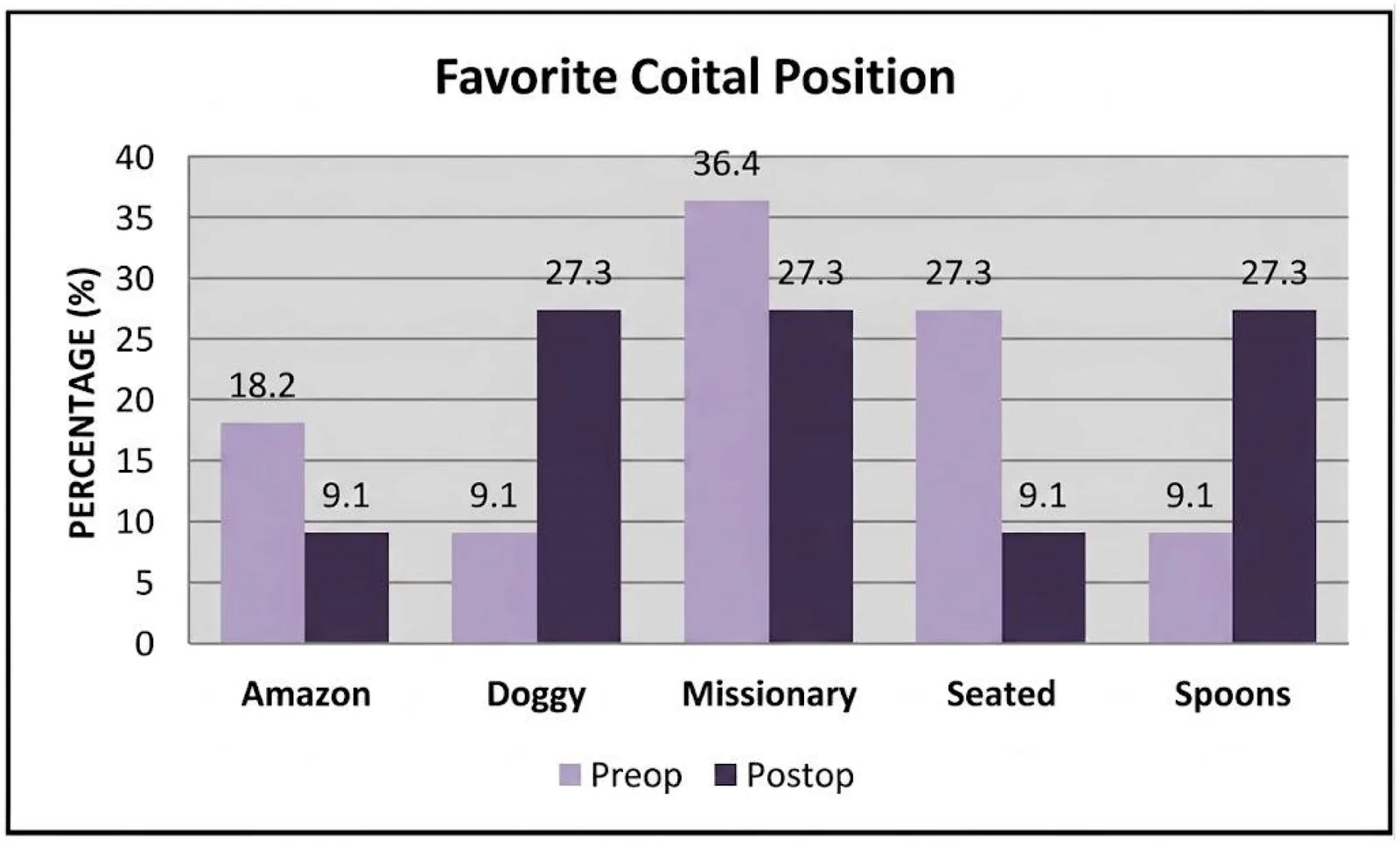
Preferred coital positions before and two years after surgery. Values are presented as the number of patients and percentages.

**Figure 6:**
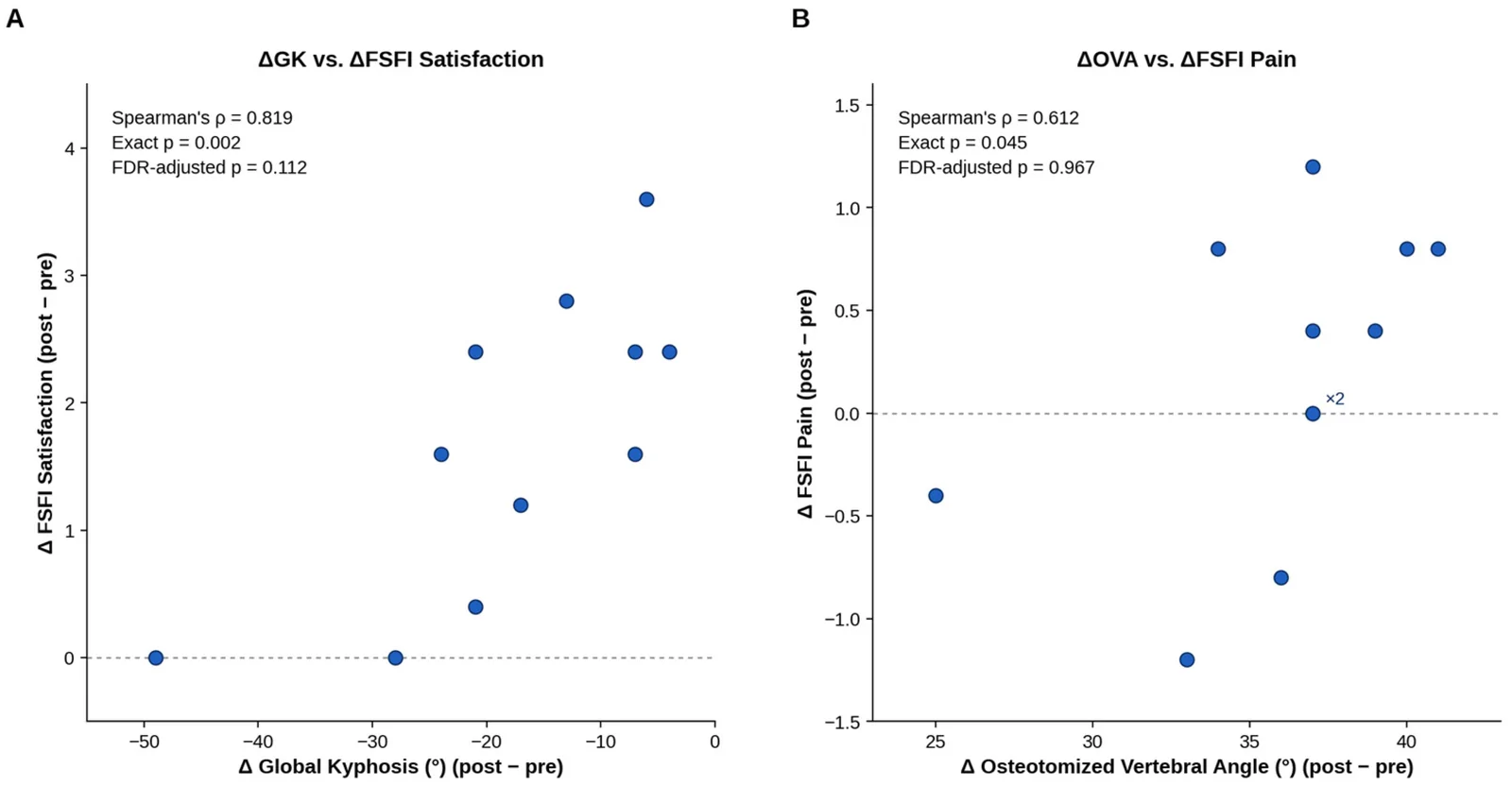
Individual-level associations between changes in FSFI satisfaction and global kyphosis (ΔGK) and between changes in the FSFI pain domain and osteotomized vertebral angle (ΔOVA) in 11 patients. Δ values represent postoperative minus preoperative changes. Spearman’s rank correlation coefficients, exact two-sided *p* values, and false-discovery-rate-adjusted *p* values are shown. Each point represents one patient; overlapping observations are indicated (×2).

**Table 1:** Demographic and clinical characteristics of the patients.

Characteristic	Statistic	Value
Age	Mean ± SD	39.27 ± 6.25
Age	Median (Min–Max)	41 (29–50)
Length of constructs	Mean ± SD	8.82 ± 2.52
Length of constructs	Median (Min–Max)	9 (5–14)
Follow-up time (month)	Mean ± SD	58.91 ± 29.28
Follow-up time (month)	Median (Min–Max)	62 (24–98)
BMI	Mean ± SD	25.57 ± 2.65
BMI	Median (Min–Max)	25.1 (20.7–29.3)
Postop Resumption of Sexual Activity (week)	Mean ± SD	7.91 ± 1.76
Postop Resumption of Sexual Activity (week)	Median (Min–Max)	8 (5–10)

BMI, body mass index; SD, standard deviation.

**Table 2:** Radiological outcomes.

		Preoperative	Postoperative	Change (Postoperative − Preoperative)	Exact *p* Value	Effect Size (r)	95% CI for Mean Change
Pelvic tilt (°)	Mean ± SD	44.00 ± 13.58	30.18 ± 8.67	−13.82 ± 7.56	0.002	−0.858	−16.82 to −7.19
	Median (Min–Max)	51 (12–59)	32 (16–43)	−16 (−21 to 6)			
Pelvic incidence (°)	Mean ± SD	51.27 ± 14.72	50.73 ± 14.30	−0.55 ± 5.47	0.700	−0.147	−3.64 to 2.45
	Median (Min–Max)	53 (25–73)	53 (26–75)	−1 (−9 to 8)			
Sacral slope (°)	Mean ± SD	7.36 ± 8.09	20.45 ± 8.48	13.09 ± 5.22	<0.001	0.885	9.36 to 15.55
	Median (Min–Max)	8 (−6–21)	20 (10–37)	14 (1–20)			
Lumbar lordosis (°)	Mean ± SD	−10.45 ± 11.12	−46.82 ± 14.34	−36.36 ± 10.38	<0.001	−0.885	−41.91 to −30.27
	Median (Min–Max)	−8 (−26–8)	−46 (−68 to −27)	−38 (−51 to −21)			
Thoracic kyphosis (°)	Mean ± SD	51.55 ± 15.86	47.55 ± 15.17	−4.00 ± 7.28	0.102	−0.523	−9.36 to −0.73
	Median (Min–Max)	52 (25–72)	43 (22–74)	−5 (−22 to 6)			
Global kyphosis (°)	Mean ± SD	82.09 ± 15.43	64.18 ± 17.61	−17.91 ± 13.13	<0.001	−0.885	−27.51 to −12.18
	Median (Min–Max)	78 (64–107)	61 (29–88)	−17 (−49 to −4)			
Osteotomized vertebral angle (°)	Mean ± SD	0.00 ± 2.83	36.00 ± 4.07	36.00 ± 4.34	<0.001	0.885	32.55 to 37.82
	Median (Min–Max)	0 (−5–5)	36 (28–42)	37 (25–41)			
Sagittal vertical axis (mm)	Mean ± SD	131.18 ± 66.29	41.82 ± 34.91	−89.36 ± 43.71	<0.001	−0.885	−114.09 to −65.82
	Median (Min–Max)	125 (39–226)	33 (−5–113)	−79 (−161 to −37)			

SD, standard deviation; CI, confidence interval. All values are based on the 11 patients with complete preoperative and two-year postoperative data. *p* values are exact two-sided values from the Wilcoxon signed-rank test; radiological parameters were secondary outcomes, and their *p* values are nominal and were not adjusted for multiple comparisons. Effect sizes were calculated as r = Z/√N (*N* = 11); 95% CIs for the mean change were estimated by bias-corrected and accelerated (BCa) nonparametric bootstrap with 10,000 resamples.

**Table 3:** Sexual activity frequency and preferred coital positions.

Outcome	Category	Preoperative, *n* (%)	Postoperative, *n* (%)
Sexual Activity (per month)	1–4 times	5 (45.5)	2 (18.2)
Sexual Activity (per month)	5–8 times	4 (36.4)	5 (45.5)
Sexual Activity (per month)	9–12 times	1 (9.1)	3 (27.3)
Sexual Activity (per month)	>12 times	1 (9.1)	1 (9.1)
Favorite Coital Position	Amazon	2 (18.2)	1 (9.1)
Favorite Coital Position	Doggy	1 (9.1)	3 (27.3)
Favorite Coital Position	Missionary	4 (36.4)	3 (27.3)
Favorite Coital Position	Seated	3 (27.3)	1 (9.1)
Favorite Coital Position	Spoons	1 (9.1)	3 (27.3)

Values are presented as *n* (%).

**Table 4:** Functional outcomes in the paired-analysis population (*n* = 11).

Outcome	Statistic	Preoperative (*n* = 11)	Postoperative (*n* = 11)	Change (Postoperative − Preoperative)	Exact *p* Value ^a^	Effect Size (r) ^b^	95% CI for Mean Change ^c^
VAS	Mean ± SD	6.55 ± 1.51	1.91 ± 1.30	−4.64 ± 1.91	<0.001	−0.885	−5.73 to −3.64
	Median (Min–Max)	7 (4–9)	2 (0–4)	−5 (−8 to −2)			
BASFI	Mean ± SD	4.38 ± 0.53	2.91 ± 0.45	−1.47 ± 0.48	<0.001	−0.885	−1.75 to −1.22
	Median (Min–Max)	4.4 (3.6–5.5)	3 (2.2–3.6)	−1.4 (−2.3 to −0.8)			

VAS, Visual Analog Scale; BASFI, Bath Ankylosing Spondylitis Functional Index; SD, standard deviation; CI, confidence interval. All values are based on the 11 patients with complete preoperative and two-year postoperative data (paired complete-case analysis). VAS and BASFI were secondary outcomes analyzed in an exploratory manner; *p* values are nominal and were not adjusted for multiple comparisons. ^a^ Exact two-sided Wilcoxon signed-rank test. ^b^ Calculated as r = Z/√N (*N* = 11). ^c^ Bias-corrected and accelerated (BCa) nonparametric bootstrap with 10,000 resamples.

**Table 5:** Female Sexual Function Index scores in the paired-analysis population (*n* = 11).

Outcome	Statistic	Preoperative (*n* = 11)	Postoperative (*n* = 11)	Change (Postoperative − Preoperative)	Exact *p* Value ^a^	Effect Size (r) ^b^	95% CI for Mean Change ^c^
Primary outcome							
Total FSFI	Mean ± SD	23.63 ± 5.45	29.94 ± 2.48	6.31 ± 3.87	<0.001	0.885	4.19 to 8.55
	Median (Min–Max)	23.9 (12.4–31.5)	30.7 (26–33.3)	6.6 (0.3–13.6)			
Secondary outcomes (FSFI domains; exploratory, nominal p values not adjusted for multiplicity)							
Desire	Mean ± SD	4.42 ± 1.38	5.89 ± 0.24	1.47 ± 1.24	0.008	0.760	0.82 to 2.24
	Median (Min–Max)	4.8 (2.4–6)	6 (5.4–6)	1.2 (0–3.6)			
Arousal	Mean ± SD	3.33 ± 1.23	4.66 ± 0.76	1.34 ± 0.88	0.002	0.858	0.87 to 1.88
	Median (Min–Max)	3.6 (0–4.5)	4.5 (3–5.7)	1.2 (−0.3 to 3)			
Lubrication	Mean ± SD	3.63 ± 1.27	3.93 ± 1.57	0.30 ± 0.84	0.275	0.353	−0.19 to 0.74
	Median (Min–Max)	3.6 (1.2–5.4)	4.2 (1.2–6)	0.6 (−0.9 to 1.5)			
Orgasm	Mean ± SD	3.42 ± 1.37	4.76 ± 0.70	1.35 ± 1.31	0.006	0.784	0.69 to 2.18
	Median (Min–Max)	3.6 (1.2–4.8)	4.8 (3.6–6)	1.2 (−0.4 to 4)			
Satisfaction	Mean ± SD	3.53 ± 1.02	5.20 ± 0.51	1.67 ± 1.18	0.004	0.804	0.98 to 2.33
	Median (Min–Max)	3.6 (1.6–4.8)	5.2 (4.4–6)	1.6 (0–3.6)			
Pain	Mean ± SD	5.31 ± 0.57	5.49 ± 0.36	0.18 ± 0.75	0.496	0.232	−0.33 to 0.55
	Median (Min–Max)	5.2 (4.8–6)	5.6 (4.8–6)	0.4 (−1.2 to 1.2)			

FSFI, Female Sexual Function Index; SD, standard deviation; CI, confidence interval. All values are based on the 11 patients with complete preoperative and two-year postoperative FSFI data (paired complete-case analysis). Total FSFI was the designated primary outcome. The six FSFI domains were secondary outcomes analyzed in an exploratory manner; their *p* values are nominal and were not adjusted for multiple comparisons. Zero differences were excluded from the signed-rank test. ^a^ Exact two-sided Wilcoxon signed-rank test. ^b^ Calculated as r = Z/√N (*N* = 11). ^c^ Bias-corrected and accelerated (BCa) nonparametric bootstrap with 10,000 resamples.

**Table 6:** Relationships between changes in radiological parameters and changes in FSFI scores.

Parameter	Statistic	ΔDesire	ΔArousal	ΔLubrication	ΔOrgasm	ΔSatisfaction	ΔPain	ΔTotal
ΔPT	r	−0.520	0.037	0.062	−0.596	−0.334	0.041	−0.389
	*p*	0.101	0.915	0.857	0.053	0.315	0.904	0.237
ΔPI	r	−0.402	0.014	−0.267	−0.550	−0.446	0.090	−0.359
	*p*	0.220	0.968	0.428	0.079	0.169	0.792	0.279
ΔSS	r	0.358	0.269	−0.494	0.105	0.124	0.147	0.302
	*p*	0.280	0.423	0.122	0.759	0.717	0.665	0.367
ΔLL	r	−0.078	−0.103	0.142	0.000	−0.158	0.323	0.050
	*p*	0.820	0.763	0.677	1.000	0.642	0.332	0.883
ΔTK	r	0.450	−0.060	−0.115	0.368	0.249	−0.023	0.217
	*p*	0.165	0.861	0.736	0.266	0.461	0.946	0.522
ΔGK	r	0.250	0.428	0.365	0.558	0.819	−0.349	0.491
	*p*	0.458	0.189	0.270	0.074	0.002 **	0.293	0.125
ΔOVA	r	0.127	0.210	−0.337	−0.154	0.058	0.612	0.231
	*p*	0.709	0.536	0.312	0.651	0.865	0.045 *	0.494
ΔSVA	r	0.085	−0.278	−0.393	−0.123	−0.466	−0.069	−0.183
	*p*	0.805	0.408	0.232	0.719	0.149	0.840	0.591

FSFI, Female Sexual Function Index; PT, pelvic tilt; PI, pelvic incidence; SS, sacral slope; LL, lumbar lordosis; TK, thoracic kyphosis; GK, global kyphosis; OVA, osteotomized vertebral angle; SVA, sagittal vertical axis; Δ, postoperative–preoperative change; r, Spearman’s rank correlation coefficient. Rows labeled “*p*” show unadjusted exact two-sided *p* values. The Benjamini–Hochberg FDR procedure was applied as a single family across all 56 correlations. The FDR-adjusted *p* values were 0.112 for ΔGK–ΔSatisfaction and 0.967 for ΔOVA–ΔPain; thus, neither nominal association remained statistically significant after adjustment. * nominal *p* < 0.05; ** nominal *p* < 0.01.

## Data Availability

The data presented in this study are available on request from the corresponding author. The data are not publicly available due to patient privacy and ethical restrictions.

## Abbreviations

AS, ankylosing spondylitis; BASFI, Bath Ankylosing Spondylitis Functional Index; BMI, body mass index; FSFI, Female Sexual Function Index; GK, global kyphosis; LL, lumbar lordosis; OVA, osteotomized vertebral angle; PI, pelvic incidence; PSO, pedicle subtraction osteotomy; PT, pelvic tilt; SS, sacral slope; SVA, sagittal vertical axis; TK, thoracic kyphosis; VAS, Visual Analog Scale.
